# Laparoscopic Management of Abdominopelvic Splenosis: A Case Report

**DOI:** 10.7759/cureus.103118

**Published:** 2026-02-06

**Authors:** Adel M Ismail, Samah S Elbasateeny, Amer Shafie

**Affiliations:** 1 Department of Surgical Oncology, Ismailia Teaching Oncology Hospital, Ismailia, EGY; 2 Pathology, King Abdulaziz University, Rabigh Faculty of Medicine, Jeddah, SAU; 3 Pathology, Zagazig University, Faculty of Medicine, Zagazig, EGY

**Keywords:** cd8, female infertility, laparoscopic management, pelvic pain, splenosis

## Abstract

Splenosis refers to the heterotopic autotransplantation of splenic tissue to various anatomical sites, most commonly following splenic trauma or splenectomy. We report a rare case of pelvic splenosis in a female patient, eight years after a car accident and splenectomy, who presented with lower abdominal pain and infertility without improvement. Laparoscopic exploration revealed ectopic splenic tissue, and the diagnosis was confirmed by biopsy. Pelvic splenosis should be considered in the differential diagnosis of unexplained pelvic or abdominal masses in patients with a history of splenic injury or surgery. Laparoscopic exploration and biopsy are very important tools for the diagnosis of abdominal splenosis.

## Introduction

Splenosis is a term denoting the focal implantation of solitary or multiple deposits formed of splenic tissue detected in different body compartments. Abdominal splenosis is caused by direct implantation of splenic deposits or blood spread of splenic tissue fragments within the compartments of the abdominal cavity [1,2].

Abdominal splenosis follows surgery such as splenectomy or trauma of the abdomen, resulting from the implantation of several splenic tissue deposits on the peritoneal cavity that acquire a new blood supply and develop splenosis. The foci of splenosis are usually detected on serosal or peritoneal covering as solitary or multiple small deposits that can grow later and become larger [1].

We report a rare case of pelvic splenosis in a female patient, eight years after a car accident and splenectomy, who presented with lower abdominal pain and infertility without improvement.

## Case presentation

**Figure 1:**
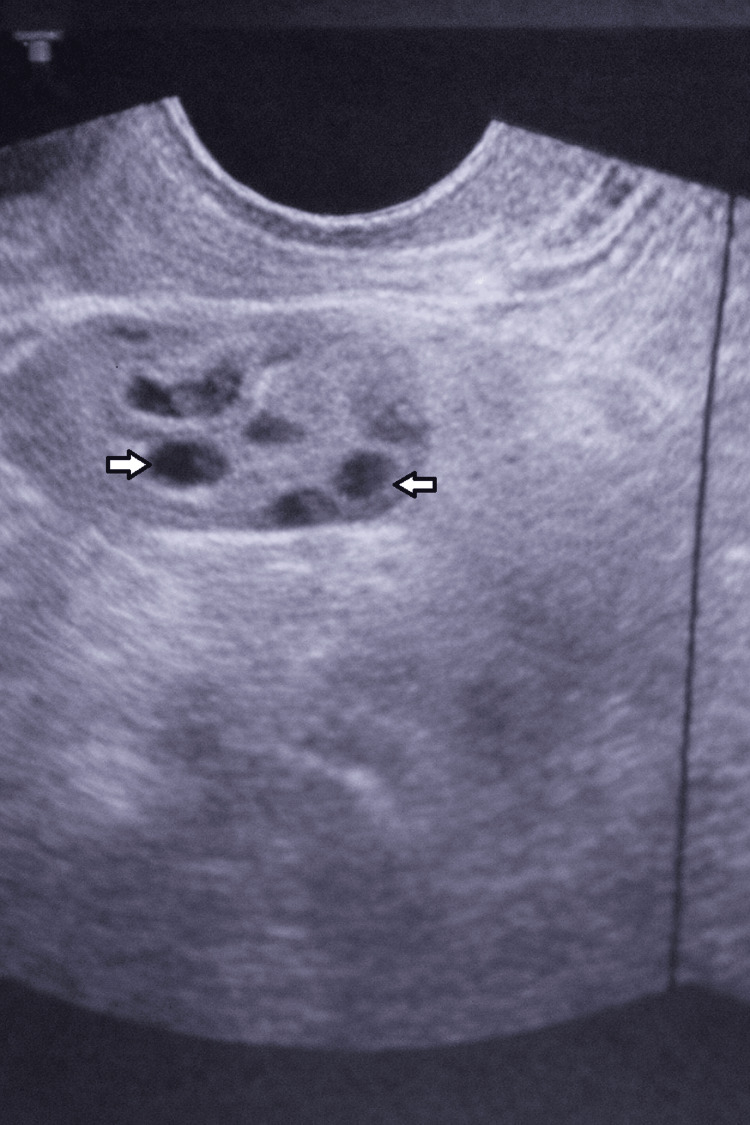
Abdominopelvic ultrasound showing multiple right adnexal masses of unknown origin

This is a case of a 31-year-old female patient with a history of a car accident eight years ago. The patient had an internal hemorrhage, multiple rib fractures, bilateral hemothorax, facial ecchymosis, and pubic bone fracture. Abdominal exploration showed a large amount of blood with a ruptured spleen. Splenectomy was done with good hemostasis and closure of the abdomen in layers. A bilateral chest tube was inserted for hemothorax. Eight years later, the patient came to the gynecological outpatient department (OPD) complaining of lower abdominal pain and lack of conception in the last two years, with no improvement. Abdominopelvic ultrasound reveals a right (RT) adnexal mass of unknown origin (Figure 1).

**Figure 2:**
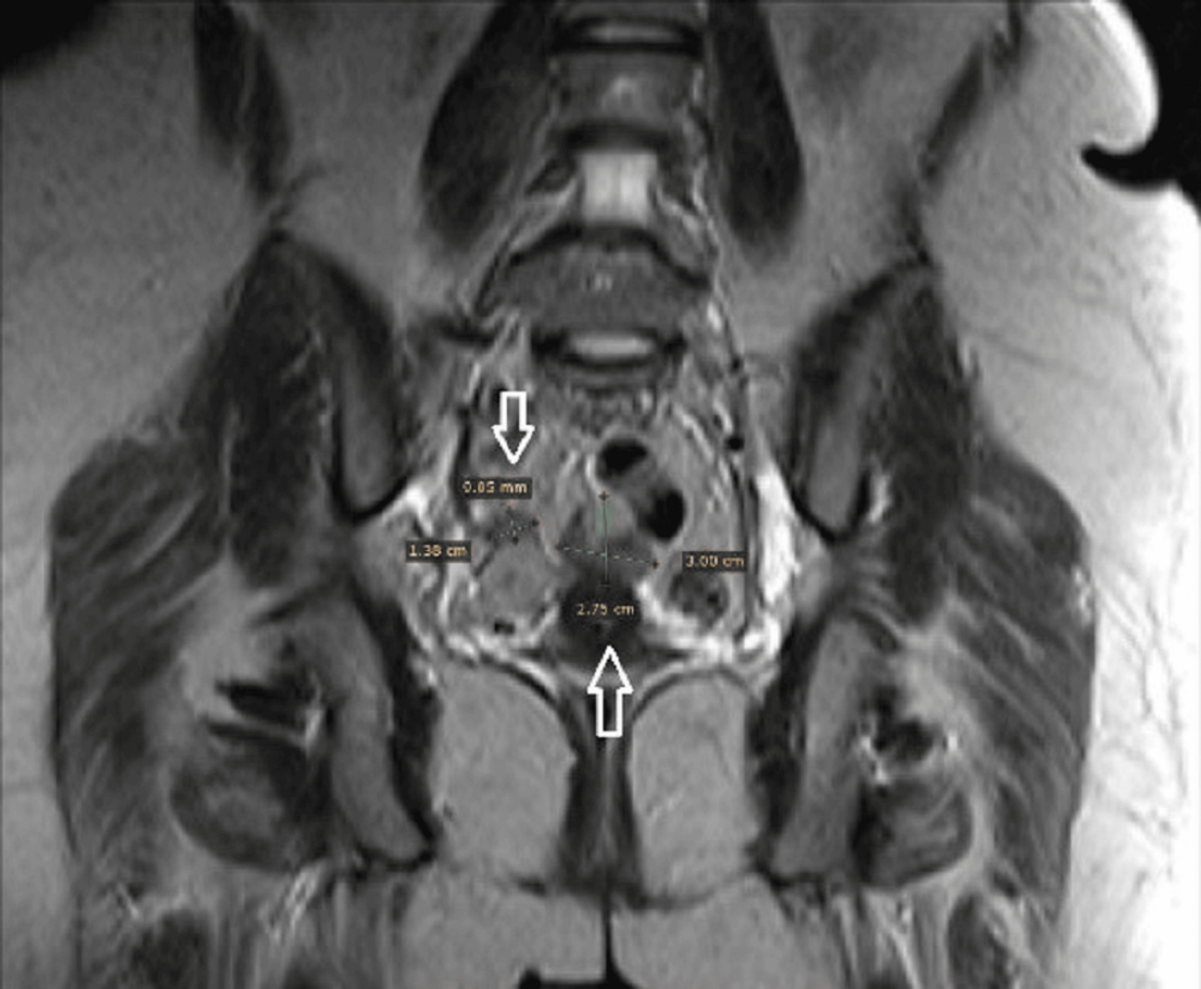
MRI showing multiple pelvic nodules (arrows)

**Figure 3:**
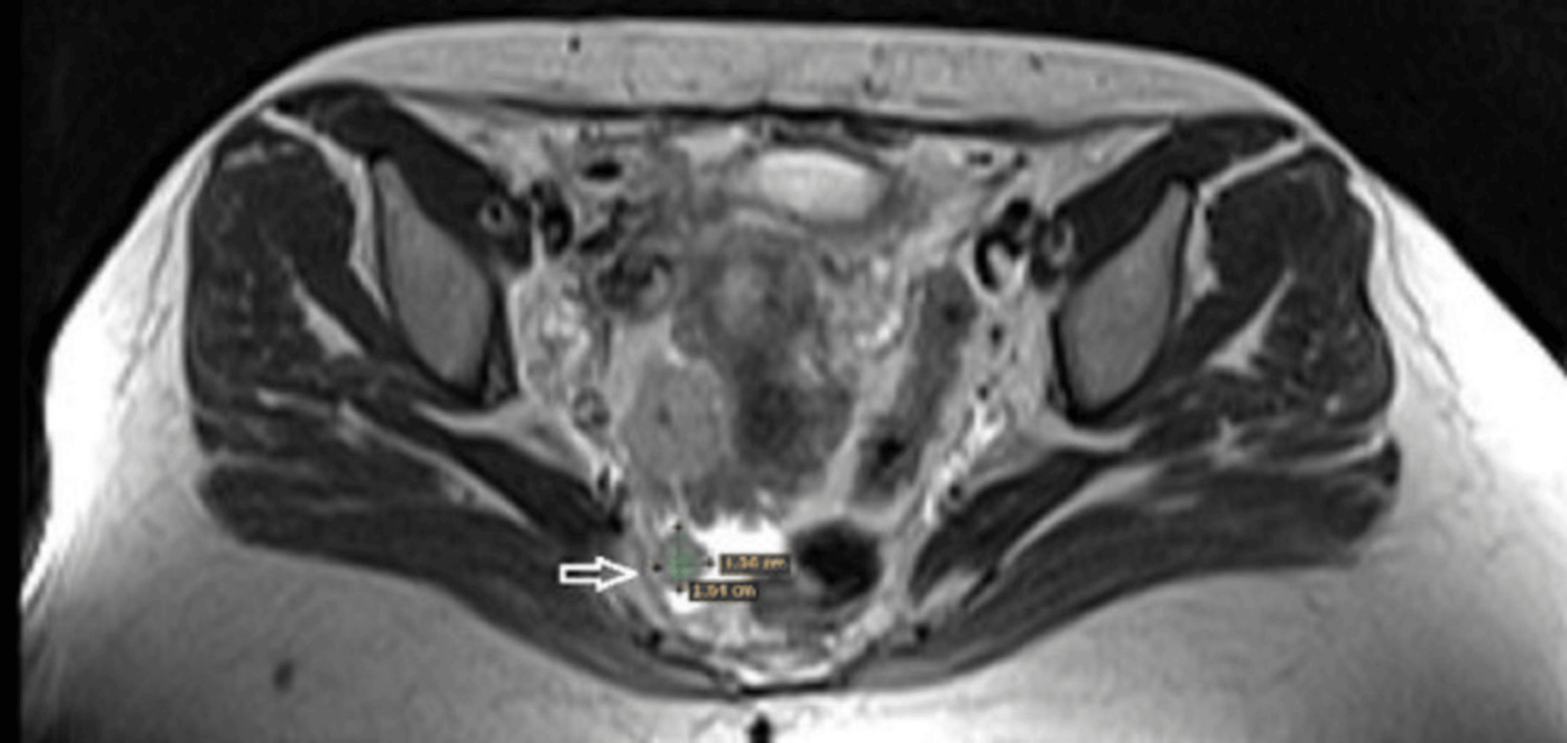
MRI showing multiple pelvic nodules (arrows)

MRI with contrast reveals multiple deeply seated variable-sized abdominal signal intensity lesions at RT side of the pelvis, recto-uterine space, and RT para uterine region. The largest measures 3.2x2.7x3.8 cm (Figures 2-3).

Differential diagnosis of MRI was pathological abdominal lymphadenopathy, splenosis, pelvic endometriosis, or bowel lesions. As there is no clear diagnosis, laparoscopic abdominal exploration was decided.

**Figure 4:**
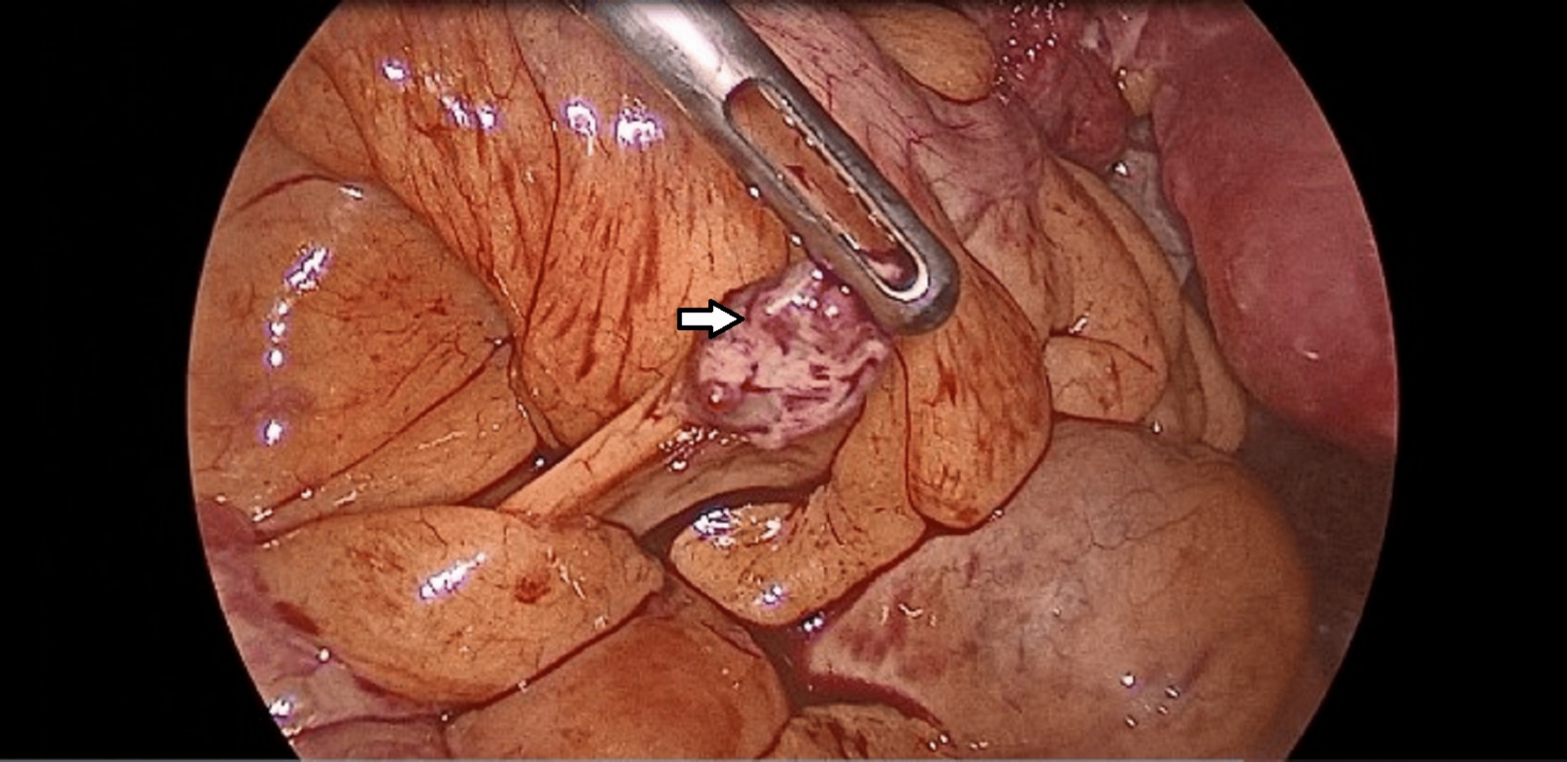
Laparoscopic image showing mesenteric splenic nodule

**Figure 5:**
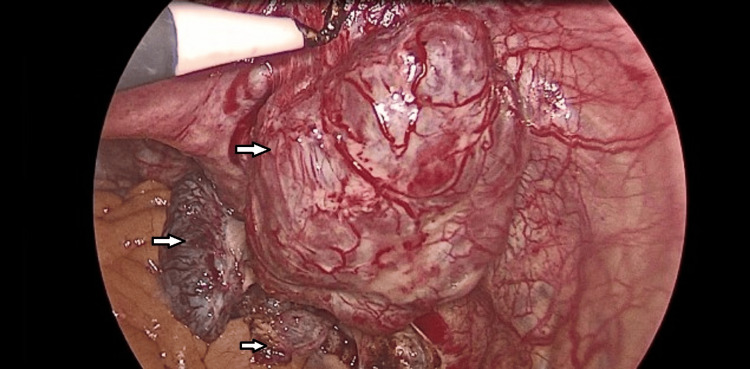
Laparoscopic image showing a large splenic nodule on the right lateral pelvic wall

On laparoscopic exploration, using a three-port technique, one camera port at the umbilicus (10 mm) and two right and left iliac ports (5 mm) away from the adhesions, multiple colonic adhesions were found as the colon was completely adherent to the anterior abdominal wall. By exploration, multiple nodules appear on the intestinal mesentery, appendix, Douglas pouch, and lateral pelvic wall with different sizes ranging from 0.5 cm to 7 cm (Figures 4-5).

Using hook diathermy, dissection and excision of the nodules at various sites of the pelvis were done. Nodules were placed in an endo-bag and retrieved from the right iliac port after wound dilatation, then the tube drain was left. On the second day, the patient was discharged after drain removal. Follow-up of the patient in the OPD for four weeks showed resolution of pelvic pain without any complications. 

**Figure 6:**
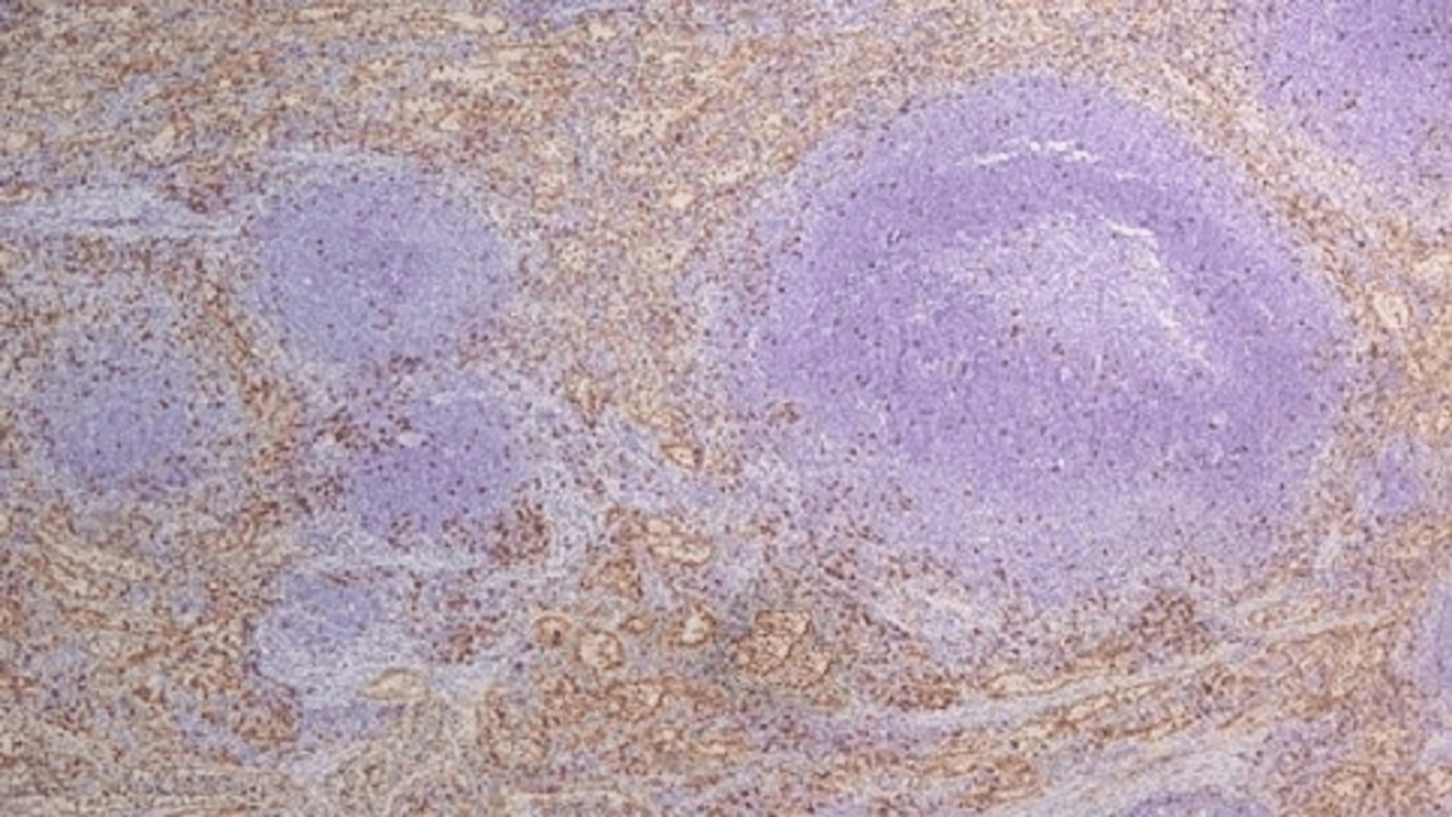
CD8 immunohistochemical staining showing positivity in the red pulp of pelvic splenosis nodules (IHC×200)

**Figure 7:**
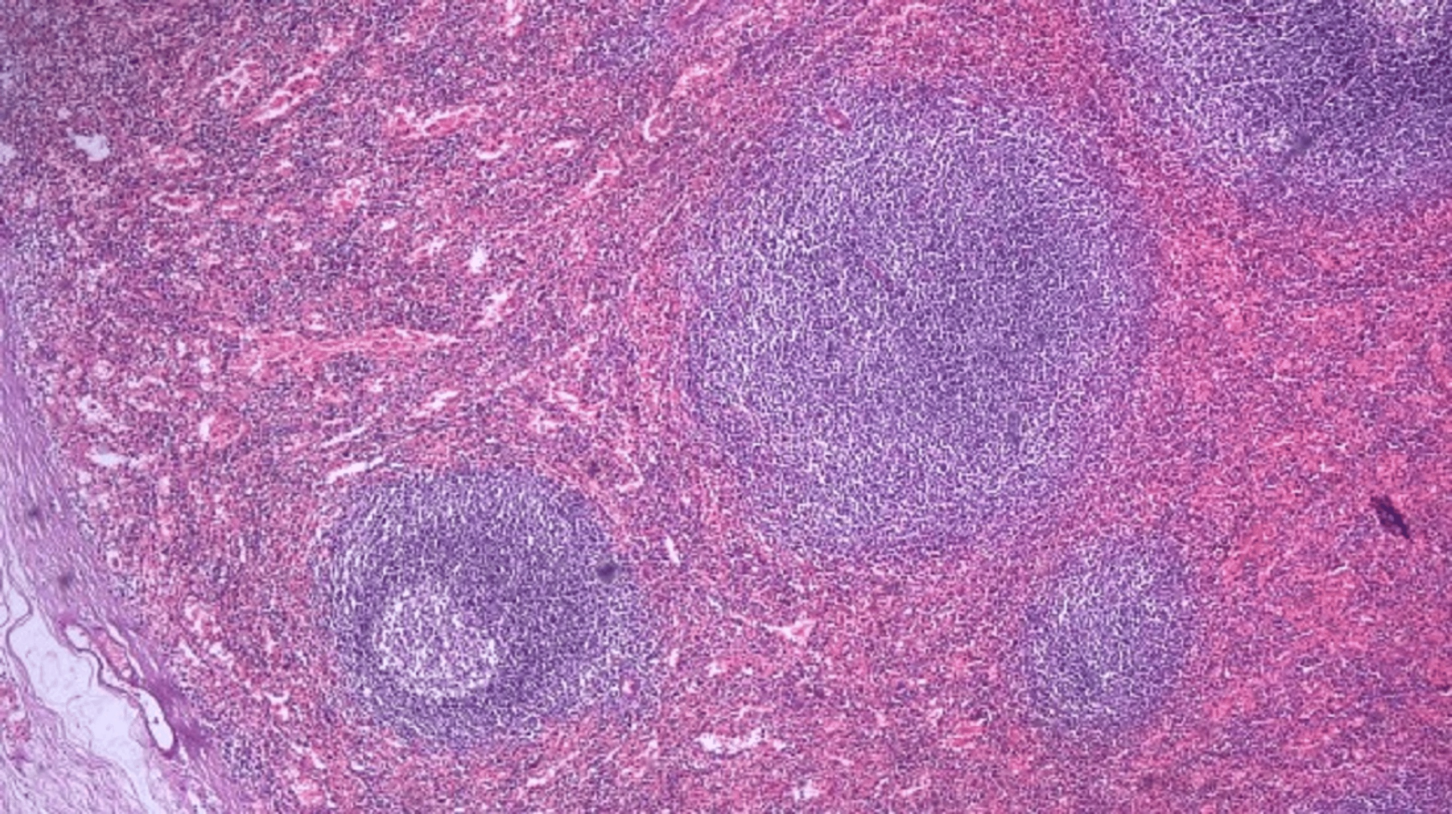
Histopathological examination of the pelvic nodules showing splenic architecture (white and red pulp, H&EX200)

Nodules were sent to the histopathology department and stained with H&E, which microscopically showed splenic tissue, which was confirmed by CD8 immunohistochemical marker staining (Figures 6-7). 

## Discussion

The term abdominal splenosis describes focal deposits of splenic tissue detected on the serosal and peritoneal covering of the abdominal and pelvic compartments. A majority of cases with a history of ruptured spleen or surgical splenectomy present later with splenosis [1].

In females, the foci of splenosis were mostly detected on most of the pelvic structures, including the uterosacral ligaments, posterior uterine wall, posterior aspect of the cervix, uterine fundus, ovaries, and in the rectouterine pouch [3,4]. 

Cases with uterine and adnexal splenosis usually present with no symptoms [5]. However, they may complain of intra-abdominal bleeding, deep pelvic pain, dyspareunia, and abnormal uterine bleeding [6]. With inadequate history taking, large pelvic splenosis with multiple peritoneal deposits may be misdiagnosed as uterine or ovarian metastasis [7]. In such cases, laparoscopic exploration and biopsy are the most accurate methods to confirm the diagnosis of female pelvic splenosis and differentiate it from malignancy [8]. Splenosis could be misdiagnosed as tuberculosis, endometriosis, lymphoma, or malignancy [9].

In our case, the etiology of splenosis was splenic rupture, which caused splenic dissemination into the peritoneal cavity via direct spread, and was reported accidentally six years after a car accident. This is confirmed by Mescoli et al., who reported that the most common cause of splenosis is splenic trauma or splenectomy, which appears months to years after [10].

In our case, the patient was asymptomatic for six years, and because of delayed pregnancy and lower abdominal pain, the patient sought medical advice. Tsitouridis et al. reported that splenosis is commonly discovered accidentally in patients, either during surgery or during assessment for another disease [11]. 

In our case, nodules were found over the serosal surface of the greater omentum, small and large intestine, mesentery, Douglas pouch, lateral pelvic wall, uterosacral ligament, and back of the uterus, with a large number and variable in size from a few millimeters to 7 cm. This corresponds with Toktas et al. in terms of the number and sites of nodules, but differs in size: they reported that nodules rarely exceed 3 cm, whereas in our case one exceeded 5 cm [12].

## Conclusions

Splenosis is an unusual but important clinical situation that must be considered in the differential diagnosis of abdominal or pelvic deposits, especially in women with a history of splenic surgery or splenic rupture. As many cases are asymptomatic and lack specific imaging criteria, splenosis may be mistaken for inflammatory or malignant neoplastic lesions, particularly metastatic deposits. This may result in diagnostic difficulties and inappropriate patient management. Laparoscopic exploration, biopsy, and histopathological diagnosis are very important, safe, and effective tools for the diagnosis of abdominal splenosis, helping to protect patients from unnecessary interventions and overtreatment. 
